# Fungal coexistence in the skin mycobiome: a study involving *Malassezia, Candida*, and *Rhodotorula*

**DOI:** 10.1186/s13568-024-01674-8

**Published:** 2024-02-20

**Authors:** Bharati Naik, Jayaprakash Sasikumar, Vishal B, Shankar Prasad Das

**Affiliations:** 1 Cell Biology and Molecular Genetics, Yenepoya Research Centre, Yenepoya (Deemed to be University), Mangalore, 575018 India; 2 Department of Dermatology, Venereology and Leprosy (DVL), Yenepoya Medical College Hospital (YMCH), Yenepoya (Deemed to be University), Mangalore, 575018 India

**Keywords:** Fungal coexistence, Acne vulgaris, Pityriasis versicolor, *Malassezia*, *Rhodotorula*, *Candida*, Recurring fungal infection, Antifungals, Personalized medicine, Mycobiome

## Abstract

Evidence of fungal coexistence in humans points towards fungal adaptation to the host environment, like the skin. The human commensal *Malassezia* has evolved, especially residing in sebum-rich areas of the mammalian body where it can get the necessary nutrition for its survival. This fungus is primarily responsible for skin diseases like Pityriasis versicolor (PV), characterized by hypo or hyperpigmented skin discoloration and erythematous macules. In this manuscript, we report a 19-year-old healthy female who presented with a one-year history of reddish, hypopigmented, asymptomatic lesions over the chest and a raised erythematous lesion over the face. Upon clinical observation, the patient displayed multiple erythematous macules and erythematous papules over the bilateral malar area of the face, along with multiple hypopigmented scaly macules present on the chest and back. Based on the above clinical findings, a diagnosis of PV and Acne vulgaris (AV) was made. Interestingly, the patient was immunocompetent and didn’t have any comorbidities. Upon isolation of skin scrapings and post-culturing, we found the existence of three fungal genera in the same region of the patient’s body. We further went on to confirm the identity of the particular species and found it to represent *Malassezia, Rhodotorula*, and *Candida*. We report how *Malassezia*, the predominant microbial resident skin fungus, coexists with other fungal members of the skin mycobiome. This study on an applied aspect of microbiology also shows how important it is to identify the fungal organism associated with skin infections so that appropriate therapeutics can be advised to avoid cases of relapse.

## Key points

- Identification of fungal species in skin infection is of utmost importance.
- In this report we show the existence of 3 fungal genera from the same infection site.
- Proper identification will help deliver appropriate therapeutics and prevent cases of relapse.

## Introduction

Skin acts as a protective barrier to prevent the entry of foreign pathogens and provides a home for the commensal microbiota. When this barrier is broken, it results in many skin disorders (Byrd et al. 2018; Tiew et al. 2020). Fungal infections contribute to many skin diseases when there is dysbiosis (De Pessemier et al. 2021; Iliev and Leonardi 2017). Among the several fungal pathogens, *Malassezia* is a prominent fungal genus infecting humans and other warm-blooded animals, mostly causing skin disorders (Das et al. 2021; Saunders et al. 2012). They are dimorphic, lipophilic, and commensal with the normal skin microbiome (Gaitanis et al. 2012). However, this commensalism can turn into a pathogenic state under the influence of certain undefined factors, which help yeast cells invade the stratum corneum and trigger the host immune system (Saunte et al. 2020). *Malassezia* is associated with many recurrent disorders, like Pityriasis versicolor (PV), Seborrheic dermatitis (SD), Atopic dermatitis (AD), Psoriasis, Confluent reticulate papillomatosis, Onychomycosis, Fungemia and is also associated with several cancers (Das et al. 2021). The prevalence of *Malassezia* species varies according to geographical locations, and *Malassezia furfur* is one of the most studied organisms among the 11 species affecting humans (Rudramurthy et al. 2014; White et al. 2014). *Rhodotorula* species belong to the division Basidiomycota and colonize plants, humans, and other mammals. It is a common environmental yeast that can act as an opportunistic pathogen and have the ability to colonize and infect susceptible patients (Wirth and Goldani 2012). *Rhodotorula mucilaginosa* is associated with cutaneous fungal infections diagnosed with retinoblastoma, especially among the immunocompromised, and is an emerging pathogen causing life-threatening infections (Capoor et al. 2014). Other *Rhodotorula* species like *Rhodotorula glutinis* and *Rhodotorula minuta* are isolated from the natural environment, and these species are responsible for diseases in humans like fungemia in patients who have received corticosteroids or cytotoxic drugs and are also associated with meningeal, skin, ocular, peritoneal, and prosthetic joint infections (Wirth and Goldani 2012). Human skin is colonized by another budding yeast, *Candida* not only resides in the skin but also contributes to several known infections. About 200 known *Candida* species, including *C*. *tropicalis*, *C*. *parapsilosis*, *C*. *orthopsilosis* commonly reside on healthy human skin (Branco et al. 2023; Kühbacher et al. 2017). Most of the *Candida* species associated with skin infections lead to thickening of the skin, hyperkeratosis, and erythema (Kühbacher et al. 2017). *Candida parapsilosis* has a wide distribution in nature and can be isolated from a variety of non-human sources, such as domestic animals, insects, soil, and marine environments. This yeast colonizes the human skin and mucosal membranes and is considered an emerging pathogen (Trofa et al. 2008). *Candida albicans* is the most prevalent opportunistic fungus, which is also commensal in the gastrointestinal and genitourinary tracts (Talapko et al. 2021). These organisms can invade the bloodstream and result in invasive candidiasis (Spampinato and Leonardi 2013; Tóth et al. 2018). Recently, *C. africana*, *C. duobushaemulonii*, and especially *C. auris* were classified as emerging pathogens that are difficult to treat because of their drug resistance (Černáková et al. 2021; Espinosa-Hernández et al. 2020).

The global burden of invasive fungal infections (IFIs) has increased due to immunocompromised patients. Systemic fungal infection diagnosis remains a challenge due to low sensitivity and long growth times. Skin microbiomes, dominated by *Malassezia* yeasts, are thought to be causative agents of skin diseases. However, the role of specific species and subtypes remains unclear and might also include novel species (Brito and Paulino 2023). New methodologies aim to improve accuracy and quickness, independent of pathogen isolation. Early and accurate diagnosis is crucial for preventing life-threatening situations. Fungal diagnostics has evolved from traditional methods to advanced non-culture-based tools, with advances in polymerase chain reaction (PCR) assays (droplet digital, high-resolution melt analysis), microfluidic chip technology, metagenomics/next-generation sequencing (NGS), biosensors, nanotechnology, and artificial intelligence (Eghtedarnejad et al. 2023; Fang et al. 2023; Jenks et al. 2023; Mendonça et al. 2022). To diagnose these fungal organisms in humans, Internal Transcribed Spacer (ITS) sequencing is a gold standard compared to other techniques. Earlier identification of fungi was done using culture-based methodologies, but for certain fungi, such as *Malassezia*, it is not helpful. Because of its special nutritional requirements and growth conditions, it was unexplored and not properly identified for a long time (Gaitanis et al. 2012; Kaneko et al. 2007). In the post-molecular era, diagnostics took a new turn in identifying variations between these organisms (Mahmoud et al. 2014b). In addition to this, fingerprinting techniques such as Pulsed-Field Gel Electrophoresis (PFGE) (Boekhout et al. 1998; Senczek et al. 1999), Random Amplified Polymorphic DNA analysis (RAPD) (Boekhout et al. 1998; Mahmoud et al. 2014b), Amplified Fragment Length Polymorphism (AFLP) (Gupta et al. 2004; Theelen et al. 2001), and Denaturing Gradient Gel Electrophoresis (DGGE) (Theelen et al. 2001) were also used in *Malassezia* diagnosis. Currently, one of the common methods is Restriction Fragment Length Polymorphism (RFLP), which is generally combined with the PCR step (PCR-RFLP) and involves enzymatic digestion of PCR amplicons of the ITS regions for *Malassezia* species identification (Gaitanis et al. 2006; Mirhendi et al. 2005). The ITS region is between the small and large subunits of ribosomal DNA and exhibits considerable genetic variation among different species of the genus *Malassezia* (Makimura et al. 2000), and PCR-RFLP offers a reliable means of characterizing the genetic fingerprints of *Malassezia* isolates for accurate classification. It also helps in gaining insights into the taxonomic diversity and evolutionary relationships with other fungi. A deeper understanding of the pathogenic potential of different *Malassezia* species in various clinical contexts is needed to understand how *Malassezia* affects humans.

Fungal co-infection is often caused by the presence of two or more fungal species in the same host (Santus et al. 2021) These infections can be difficult to diagnose and treat and can be more severe than single fungal infections (Mohandas and Ballal 2011; Santus et al. 2021). Several factors contribute to the infection spread, including immune suppression and environmental-related ones (Zhao et al. 2021). Also, host genetics play an important role in the development of fungi-associated diseases and their progression (Naik et al. 2021). Fungal co-infections are rare but not uncommon, mostly found in immunocompromised patients but also reported among normal patients (Ahmed et al. 2016). These yeasts are present in their budding form and often transform themselves into hyphal forms, which are associated with infection (Boyce and Andrianopoulos 2015). Investigations have revealed that fungi-fungi or fungi-bacteria coexistence can occur simultaneously in an individual who might not necessarily have an immunocompromised condition (Arvanitis and Mylonakis 2015; Blaize et al. 2020; Pate et al. 2006). Skin diseases associated with fungal infection are usually treated with antifungals, and in cases where there is an associated inflammatory skin condition, it is often supplemented by anti-inflammatory therapy (Prohić et al. 2016; Saunte et al. 2020; Theelen et al. 2018). Examples of fungal coinfections include Mucormycetes (Zygomycetes) and *Aspergillus*, reported in patients post-Covid infection (Vadher et al. 2023), *Pneumocystis jirovecii* and *Aspergillus fumigatus* in non-HIV immunocompromised patients (Markantonatou et al. 2017), *Cryptococcus neoformans* and *Aspergillus* in an adult male with no underlying chronic disease or immune deficiency (Lamps et al. 2014), *Rhodotorula mucilaginosa* and *Trichosporon asahii* in the infected toe nail onychomycosis (Idris et al. 2020) and intra-genus co-infection among *Malassezia* species (Dyląg et al. 2020). Other studies have focused on the co-infection of fungi with bacteria (Krüger et al. 2019) (Grimshaw et al. 2019). Works on *Malassezia* co-infection are limited to a few studies, mainly because of its difficulty in culturing and maintenance, but in the case of *Candida*, there have been a handful of studies on its co-infection with bacteria. All these reports highlight the potential risks of human exposure to these fungi and fungal diseases as a result of dysbiosis and the importance of early diagnosis and treatment.

The possible treatment mechanisms for *Malassezia* infection include anti-fungal agents such as Ketoconazole, Clotrimazole, Voriconazole, Itraconazole, Posaconazole, and Econazole, which are commonly used for localized infections, either topical or systemic (Leong et al. 2017; Wang et al. 2020). They inhibit the synthesis of Ergosterol, a key component of fungal cell membranes. Another antifungal, Terbinafine acts by disrupting cell membrane integrity (Gupta and Kohli 2003). Often, antifungals are prescribed without knowledge of the causative fungus behind the infection, and this often leads to relapses of infections because of their inappropriate uses. However, in contemporary medical research, there has been a discernible shift towards exploring alternative strategies to mitigate the risks associated with azole-based drugs in the treatment of *Malassezia*-related infections. This shift is particularly evident in the growing significance of plant extract-based studies as promising alternatives. Plant extracts have emerged as compelling candidates, harnessing the therapeutic potential of natural compounds to combat *Malassezia* while minimizing the drawbacks (potential side effects) associated with synthetic drugs. Noteworthy examples include the exploration of tea tree oil, known for its antifungal properties (Sibi 2023), and extracts from fenugreek, neem, aloe vera, and dill seed (Gebremedhin et al. 2020; Mahmoud et al. 2014a; Ogbeba 2020; Panicker et al. 2020), all of which are used for their antifungal activities.

## Materials and methods

### Clinical diagnosis and sample collection

**Fig. 1:**
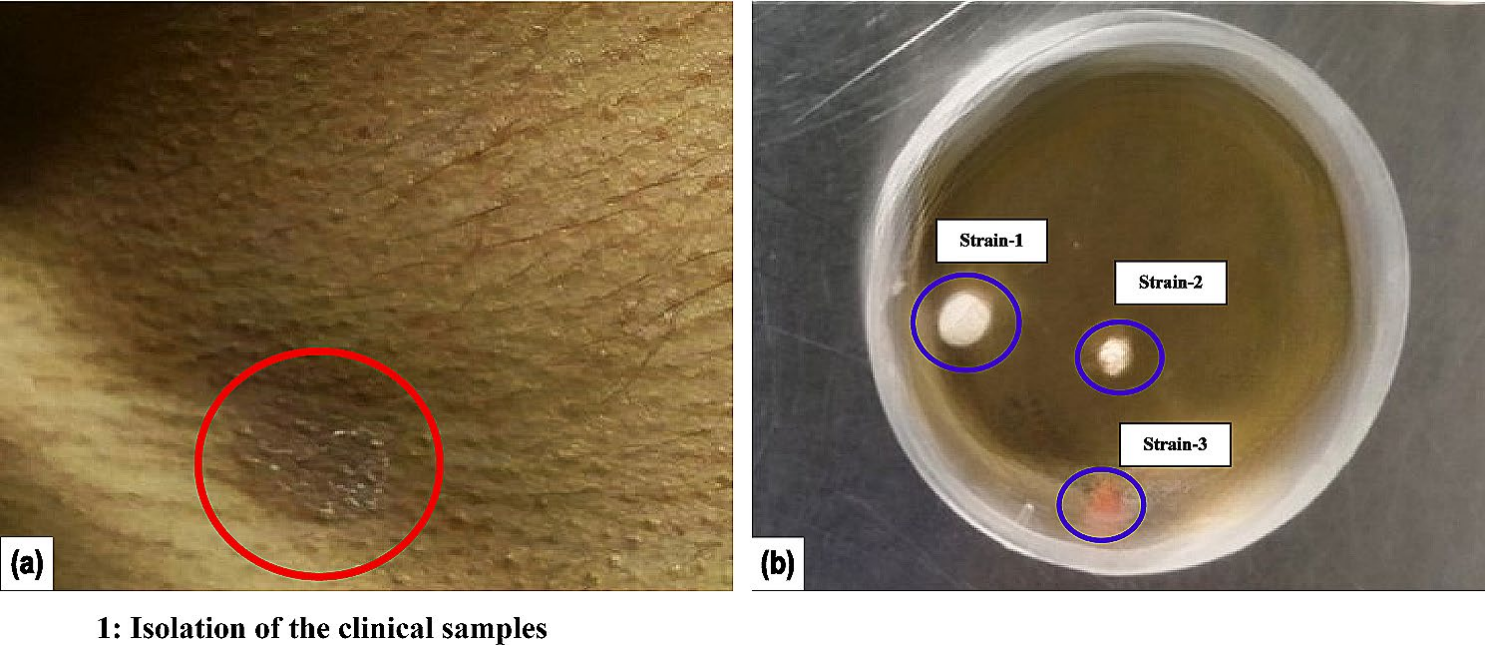
Isolation of the clinical samples. **a** Image of the infected region of the patient. **b** Growth of the clinical sample from the incubated skin scrapings

A 19-year-old healthy female presented with a past one-year history of reddish hypopigmented asymptomatic lesions over the face and chest at the Department of Dermatology, Venereology, and Leprosy, Yenepoya Medical College Hospital (YMCH), Mangalore, India. On observation, the patient revealed multiple erythematous papules and a macular erythematous scar over the bilateral malar area of the face. Upon further observation, multiple hypopigmented scaly macules were seen to be present over the chest and back (Fig. 1a). The patient was advised to take Fluconazole, Minocycline, sunscreen, and Benzoyl peroxide. Following an episode of relapse, the patient came for a follow-up visit after one month with improvement along with new lesions. On examination, multiple erythematous papules were present over the bilateral malar area of the face, and multiple scaly hypopigmented and hyperpigmented macules over the chest and upper neck. The case was diagnosed as AV and PV. Clinicians advised Isotretinoin, Fluconazole, Clotrimazole, Salicylic acid 30% peel, and Candid TV shampoo. After the next three months, in the follow-up visit, the patient reported multiple reddish lesions over the face. On observation, multiple erythematous grouped solitary papules and a few pustules over the bilateral malar and the forehead were observed. Minocycline, vitamin D gel, and Salicylic acid 30% peel were advised. The scrapings of the patients were collected as per the standard protocol (Kurade et al. 2006) with proper informed consent. The study was approved by the Yenepoya University Ethics Committee Protocol Number YEC-1/2019/018.

### Isolation of fungal specimens

The collected scrapings were brought to the lab and incubated in modified Dixon’s agar (mDA, HiMedia) supplemented with Chloramphenicol (50 µg/ml) at 32ºC until visible colony growth was observed. *Malassezia furfur* 1374 (control strain MC) was obtained from the Microbial Type Culture Collection (MTCC), Chandigarh, India.

### Purification & glycerol stocks

**Fig. 2:**
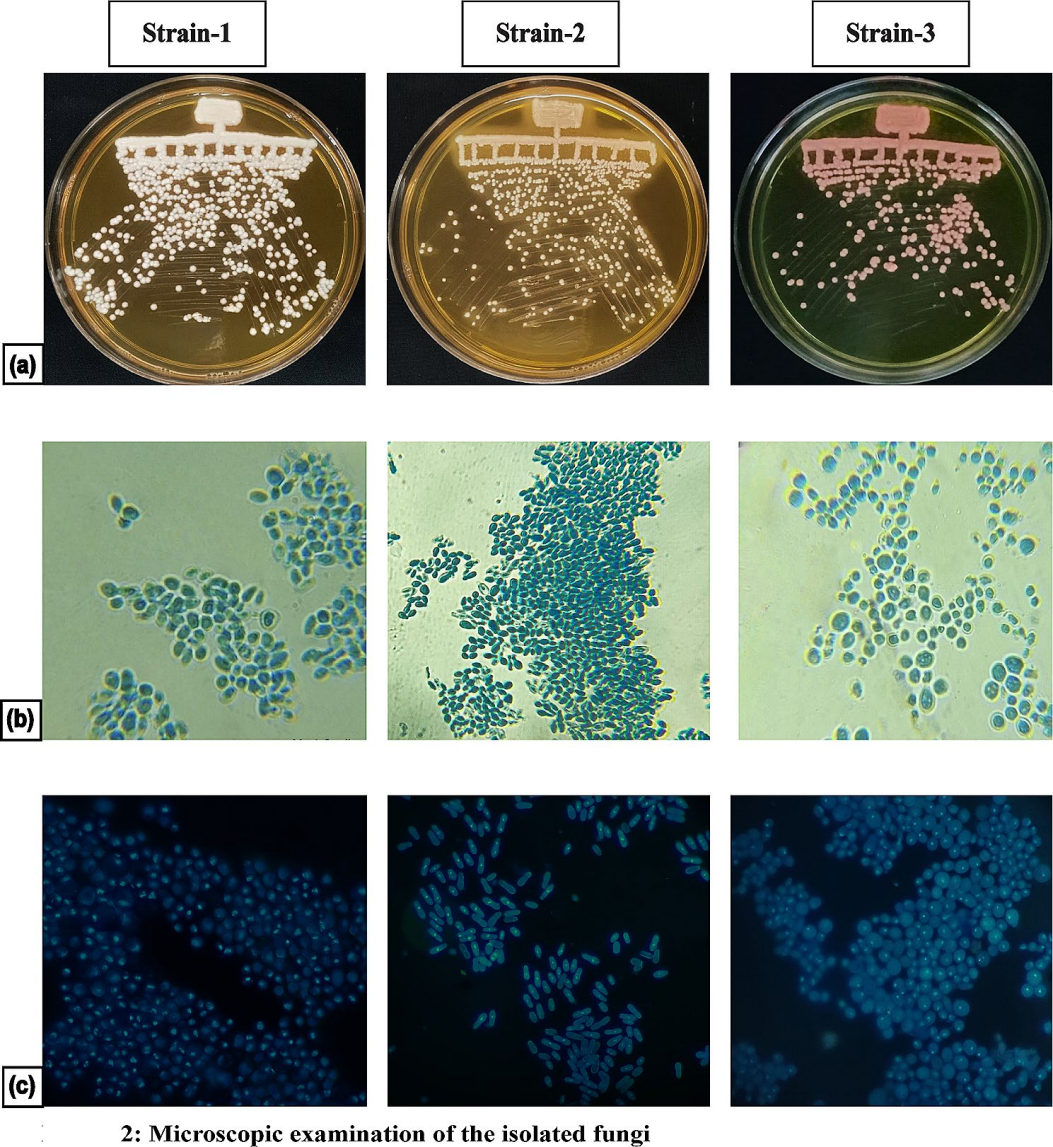
Microscopic examination of the isolated fungi. **a** Purified culture obtained from the incubated skin scrapings. **b** Lactophenol cotton blue staining of the yeast. **c** DAPI staining of the isolated yeast cells

The colonies were carefully purified from the mother plate and streaked for single colonies (Fig. 2). After purification, the cultures were stored at -80ºC in 20% glycerol stock for future use.

### Staining of the cells and nucleus

The purified yeast cells were stained with Lactophenol Cotton Blue (LPCB), HiMedia. A drop of the stain was placed on a clean, dry slide with coverslip, waited for 5 min and then observed under the microscope. Nuclear staining was performed using 4′,6-diamidino-2-phenylindole (DAPI) (Sigma). Modification of the DAPI protocol was done for staining *Malassezia* (unpublished), which is otherwise difficult to stain due to the thick cell wall.

### Culture media

To study various features of these organisms, we grew the yeasts in liquid Dixon’s media and maintained the cultures in mDA plates. Further, we performed biochemical characterization for preliminary identification of the strains (Elshabrawy et al. 2017).

### DNA isolation, PCR, RFLP, and gel electrophoresis

Fungal cultures were grown in liquid Dixons media, and using the fungal DNA isolation kit, DNA was isolated according to the manufacturer’s instructions (Norgen). The isolated DNAs were quantified using a nanodrop (Titertek Berthold). Forward ITS3 primer (GCATCGATGAAGAACGCAGC) and reverse ITS4 primer (TCCTCCGCTTATTGATATGC) were used for PCR. The PCR conditions were as follows: 95ºC for 3 min, followed by 95ºC for 1 min denaturation, 52ºC for 1 min annealing, and 72ºC for 1 min extension for 40 cycles, and the final step at 72ºC for 10 min. The PCR product was subjected to gel electrophoresis using 1.5% agarose, and an image was obtained using a gel documentation system (Vilber). PCR-RFLP using the restriction enzyme *AluI* from NEB was performed to look into the banding pattern specific to a particular species (González et al. 2009; Jagielski et al. 2014).

### Sequencing

For further identification and confirmation, DNA samples were sent to HiMedia for sequencing of the fungal ITS region. The purified PCR DNA was amplified using ITS1 and ITS4. Using the ITS1 primer, single-pass Sanger sequencing was performed for species confirmation. The sequencing results were analyzed using NCBI BLAST, and the results are presented in Table 1. The sequences obtained was submitted to GenBank, and accession numbers were obtained.

**Table 1:** BLAST analysis of nucleotide sequence of the PCR products obtained in amplification with ITS 1 primer

Identified Species		NCBI Genome ID.	Sequence length(bp)	Query Cover%	Identity %	GenBank accession number
*Rhodotorula mucilaginosa*		36242	589	98	99	OR214976
*Candida parapsilosis* (CC)		930	483	85	99	OR243746
*Malassezia furfur* (MC)		39877	681	97	97	OR243747
**Primers used in this study**						
ITS 1	5’-TCCGTAGGTGAACCTGCGG-3’					
ITS 3	5‘-GCATCGATGAAGAACGCAGC-3’					
ITS 4	5‘-TCCTCCGCTTATTGATATGC-3’					

## Results

### Isolation and purification of the clinical samples

Skin scrapings were obtained from the patient’s neck region, as indicated in (Fig. 1a). After a few days of incubation at 32ºC on the mDA plates with chloramphenicol, we observed three distinct types of colonies (Fig. 1b) and these colonies were purified and subjected to microscopic examination. Isolated colonies differed in size, shape, texture, and color and are indicated as strains 1, 2, 3 (Fig. 2a). While strain 1 was whitish, strain 2 was pale yellow, and strain 3 was pinkish.

### Staining of the purified yeast

Purified yeasts were stained with LPCB. Morphologically, Strain 3 was distinctly bigger than Strain 1 and Strain 2. Strain 2 was a bit elongated, and Strain 1 and 3 were round (Fig. 2b) as shown in the stained images of the isolated fungi. DAPI staining of the cells was performed to look into the nuclear morphology (Fig. 2c).

### Biochemical characterization

**Fig. 3:**
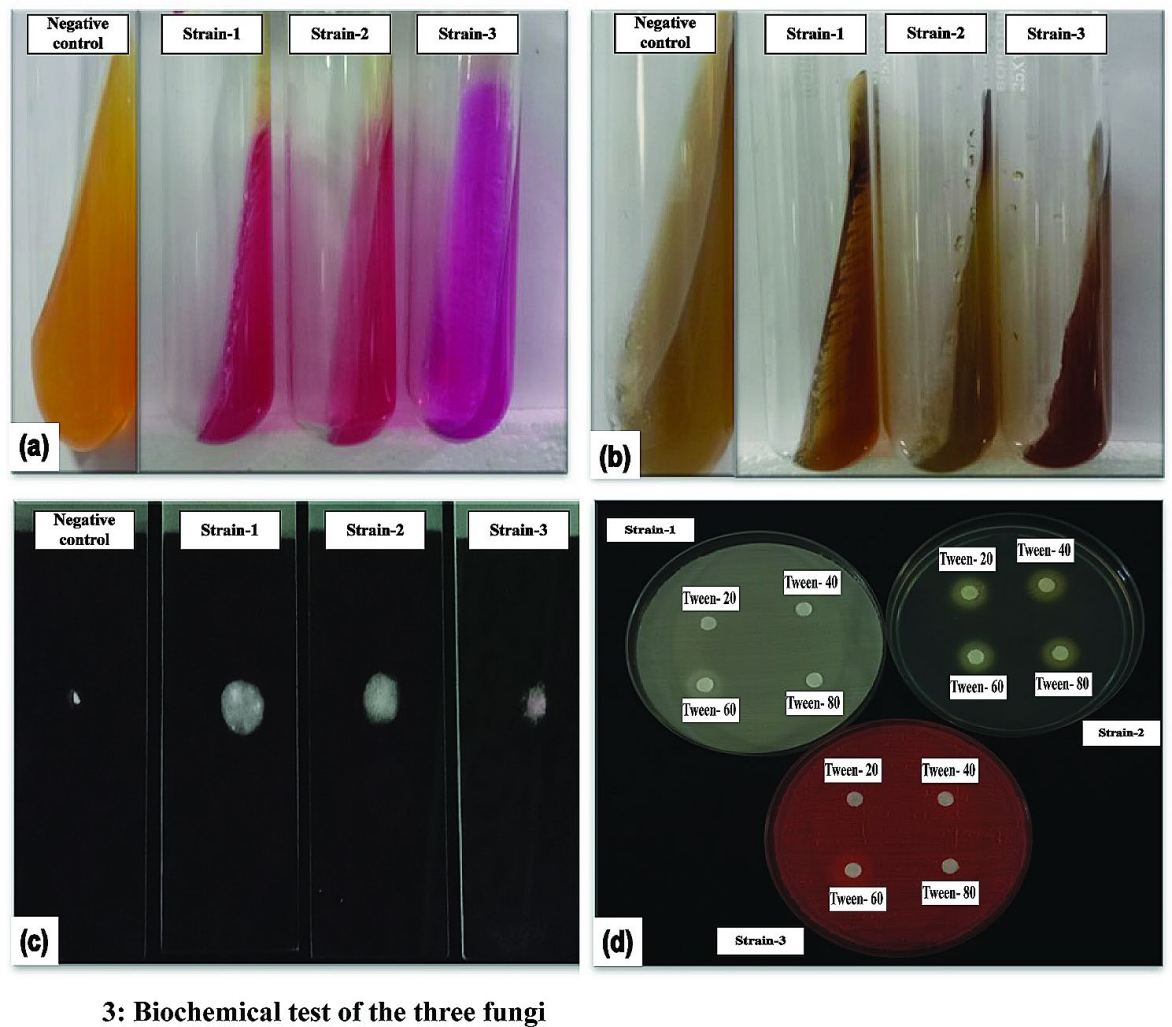
Biochemical test of the three fungi. **a** Urease Test. **b** Bile Esculin test. **c** Catalase test. **d** Tween assimilation test. NC: Negative Control

For preliminary identification of the isolated fungi, we subjected them to biochemical assays including catalase, urease, bile esculin, and tween assimilation tests. All the organisms showed similar results (Fig. 3), except for the tween assimilation test, in which only one strain (strain 2) showed clear zones of utilization for tween.

- Urease test involves the development of a pink color in the media which is indicative of the ability of the organism to split urea through the production of the enzyme urease and release ammonia and carbon dioxide (Fig. 3a).
- Bile Esculin agar test shows darkening of the media at the top of the test tubes, indicating the ability to hydrolyze esculin to esculetin, which reacts with Fe3 + and forms a dark brown to black precipitate (Fig. 3b).
- Catalase test involves bubble formation and is indicative of the presence of the catalase enzyme, which mediates the breakdown of hydrogen peroxide into oxygen and water (Fig. 3c).
- Tween assimilation tests were performed to analyze the microorganism’s ability to utilize Tween. This test is used for specific identification of lipid-dependent microorganisms like *Malassezia* which vary in their ability to utilize tween among the species (Fig. 3d).

### PCR-RFLP and sequencing

**Fig. 4:**
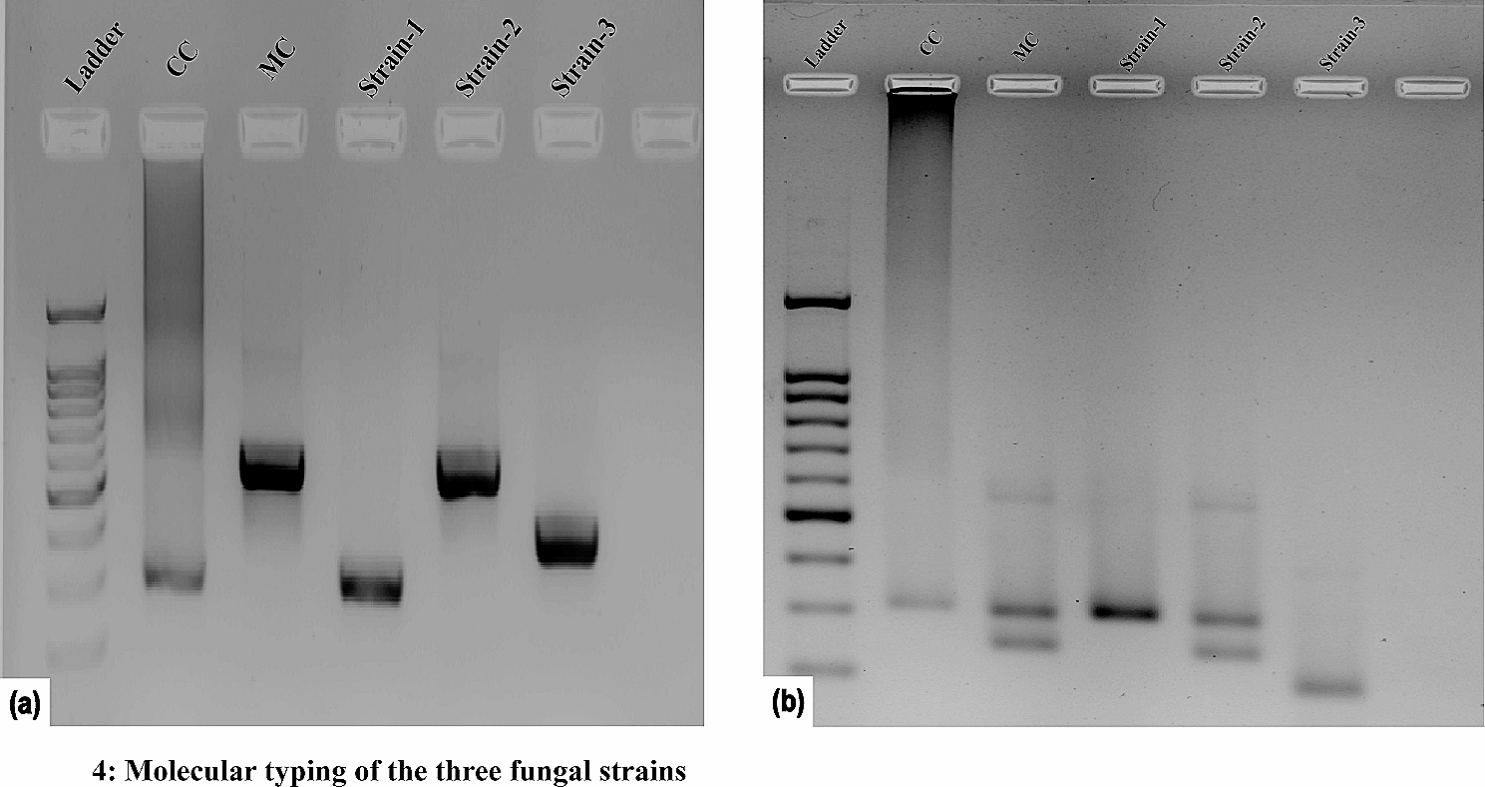
Molecular typing of the three fungal strains. **a** PCR amplification of the ITS region using primers ITS3 and ITS4. **b** RFLP of the PCR purified product using *AluI*. CC- *Candida parapsilosis* control. MC- *Malassezia furfur* control

From the PCR using the ITS primers (forward ITS3 and reverse ITS4) Table 1, we obtained three different band sizes (approx. 300 bp, 550 bp, and 350 bp), among which two strains matched our reference strains (Fig. 4a). Further RFLP results confirmed the two strains as *Malassezia furfur* (Strain 2) and *Candida parapsilosis* (Strain 1) when compared with MTCC 1374 (designated as MC) and CC (confirmed *Candida parapsilosis)* respectively (Fig. 4b). Sequencing of Strain 3, whose RFLP pattern didn’t match with the controls, turned out to be *Rhodotorula mucilaginosa.* (Fig. 4b; Table 1). Sequencing results for all three strains are shown in Table 1.

## Discussion

Our skin is the largest organ of the body and protects us against harsh environments. It is inhabited by diverse organisms that are primarily commensals and, over time, have adapted to the host microenvironment for their survival (Smythe and Wilkinson 2023). This skin microbiome is also a dynamic environment where there are interactions among the resident microbes, which include bacteria, fungi, and viruses (Byrd et al. 2018; Tiew et al. 2020). They thrive in the skin microenvironment but usually don’t harm the host unless there is dysbiosis or factors like pH, temperature, humidity, etc. that trigger alteration of the microbial balance or a condition in which the microbes take advantage of the suppressed immune status of the host. One crucial aspect of microbial infection also depends on the host’s genetics (Naik et al. 2021), which explains why some of us are more prone to fungal infections than others. Most of the studies on the human microbiome are concentrated on bacteria for infection-related studies, but current research points towards the involvement of fungi in skin-related pathogenesis. Common fungal infections, primarily caused by dermatophytes, occur in both healthy and immunocompromised patients, with a 10–20% lifetime risk (Garg et al. 2009) and a global incidence of 20–25% in healthy individuals (Burstein et al. 2020). They feed on keratin and cause various skin diseases, collectively known as dermatophytosis or tinea (Moskaluk and VandeWoude 2022; Weitzman and Summerbell 1995). It can cause symptoms such as rashes, scaling, itching, inflammation, and hair or nail loss and can be transmitted from person to person, from animal to person, or from soil to person, depending on the type of dermatophyte involved (Chanyachailert et al. 2023). They can also cause severe infections in patients with solid organ transplants or congenital immune disorders (Monod and Lanternier 2022). Apart from dermatophytes, some important commensal fungi associated with the human skin involve primarily *Candida* and *Malassezia*, but there is also the presence of several others that impact human health and necessitate greater attention to the human microbiome (Underhill and Iliev 2014; White et al. 2014). Fungi have been traditionally ignored since most of them cause diseases that can be taken care of by administering antifungals and also due to the difficulty of culturing some yeast, like *Malassezia.* How a dynamic equilibrium in the form of symbiosis is maintained by these fungal organisms is a subject of great interest among researchers (Hall and Noverr 2017) and it is important to acknowledge the risks of IFIs, particularly in immunocompromised individuals, where the disruption of the skin microbiome’s dynamic equilibrium (dysbiosis) can create opportunities for fungal pathogens to cause severe and potentially life-threatening conditions. In this report, we obtained one clinical case that shows the cohabitation of three fungal genera. Such a state is usually rare and complicates therapeutics since, in such cases, antifungal treatment might be different and medications might not be effective, leading to episodes of relapse (Dyląg et al. 2020). Hence, it is of utmost importance to identify the fungal species behind the infection and treat them accordingly.

Acne vulgaris and Malassezia folliculitis (MF) are difficult to distinguish because they can often coexist (Jakhar et al. 2019; Paichitrojjana and Chalermchai 2022; Sun and Chang 2017). These infections present as papules, pustules, nodules, and cysts on the skin (Parać et al. 2023) but MF presents with monomorphic lesions associated with pruritis (Rubenstein and Malerich 2014). In this study, a 19-year-old female was diagnosed with AV and PV based on the site of infection, skin morphology, and pruritis. The patient did not have any underlying immunodeficient conditions or any history of other skin diseases, such as MF, or acne, and was otherwise healthy. The patient had no prior history of topical corticosteroid use, which can modulate the immune system and influence the fungal diversity in the applied area. Interestingly, some of these steroids have an impact on causing and worsening the PV condition (Brandi et al. 2019; Tatnall and Rycroft 1985). Another inducing factor for PV is climate. The patient resides in one of the tropical regions of India. PV has been reported to exhibit a higher prevalence in regions with warm temperatures. Its prevalence varies and is dependent on a variety of ecological, occupational, and socioeconomic factors. Warm, humid weather also encourages the growth of *Malassezia*, which leads to summertime flare-ups of SD (Parker et al. 2022). This suggests that a warm climate and factors like host genetics may be potential contributing factors to the manifestation of recurrent *Malassezia* infection in the presented patient.

In India, *Malassezia sympodialis*, *Malassezia globosa*, and *Malassezia furfur* are the prevalent *Malassezia* species (Archana et al. 2015; Ray (Ghosh) et al. 2019; Remya et al. 2017). Among *Candida* species, *Candida albicans* and *Candida tropicalis* are the most prevalent (Gupta et al. 2015; Umamaheshwari and Sumana 2023), whereas there are no studies on *Rhodotorula* prevalence from India; however, incidence of *Rhodotorula* is estimated to be around 0.5 to 2.5% in western countries (Duboc De Almeida et al. 2008). Here, we wanted to investigate the fungi associated with the skin and found the presence of three fungal genera, namely *Candida, Malassezia*, and *Rhodotorula*, in the same infection site. We confirmed the genus initially by classical identification procedures like microscopic observation and biochemical tests and later confirmed the species by PCR-RFLP and sequencing using ITS primers. *Rhodotorula mucilaginosa* is an important pathogen, mostly affecting immunocompromised individuals, causing fungemia, central nervous system infections, peritoneal dialysis-associated peritonitis, keratitis, and also being associated with skin and soft tissue infections (Ioannou et al. 2022). In another study, a skin biopsy with the diagnosis of specific vasculitis in the upper dermis indicated the presence of *Rhodotorula* (Coppola et al. 2015). *Candida parapsilosis* is a commensal organism of human skin and is considered to be an emerging pathogen (Gácser et al. 2007). It can often be isolated in patients with vaginitis, skin lesions, and nail infections (Jautová et al. 2001). *Malassezia furfur* is commensal with the skin, and it is most common in oily areas such as the face, scalp, and back (Thayikkannu et al. 2015). This yeast causes hypopigmented lesions in cases of PV where it converts to its pathogenic filamentous form and causes damage to melanocytes (Leung et al. 2022). The characteristics of these fungal organisms are summarized in Table 2. Our study again highlights how several fungi with different nutritional requirements can coexist in the same human microenvironment and share the same niche. These emerging fungal pathogens develop resistance to antifungals, and elucidating the mechanisms behind the drug resistance will help in the identification of new therapeutic targets (Fisher et al. 2022; Revie et al. 2018). Hence, identification of the fungal culture is important for appropriate therapeutics and preventing cases of relapse. Identifying coinfections is a big challenge, mostly because of the difficulty in culturing the associated fungus (Dupuy et al. 2014). In most cases, coinfections are undetected, so patients don’t receive the appropriate medications. In this study, we have shown the presence of three fungal genera from the same region of skin infection, and this is probably the first report involving *Malassezia, Candida, and Rhodotorula.* We conclude that proper identification is important for successful treatment of such cases and preventing cases of relapse.

**Table 2:** Comparative analysis of the three fungal cohabitants

Characters	*Candida parapsilosis*	*Malassezia furfur*	*Rhodotorula mucilaginosa*
Fungal Division	Ascomycota	Basidiomycota	Basidiomycota
Genome size and Chromosome number	26 Mb 14	13.5 Mb 7	20 Mb 10
Preferred media for growth	Sabouraud Dextrose Agar/ Yeast Extract–Peptone–Dextrose	modified Dixon’s agar/ Modified Leeming and Notman agar medium	Potato Dextrose Agar/ Yeast Extract–Peptone–Dextrose
Preferred pH and temperature for growth	4–5 30–37ºC	7–9 32–35ºC	5 22–30ºC
Doubling time in rich media	Approx. 140 min	Approx. 170 min	NA
Nutritional requirement	high glucose and lipid concentrations	Fatty acid supplements	Carbohydrates and trace elements
Prevalence	Both non-human source and commensal with human	Sebum rich area, commensal with human.	Environmental and commensal with humans
Diseases associated with humans	Skin infections like erythema, Hyperkeratosis, onychomycosis, fungemia, arthritis, endocarditis, meningitis, peritonitis, urinary tract infection, vulvo-vaginitis, ocular infections	Skin disorders like pityriasis versicolor, Seborrheic dermatitis, Dandruff, Atopic dermatitis	Bloodstream infections associated with central nervous catheter
Virulence factors	Adherence, biofilm formation, protease production, phospholipase activity, and secreted aspartyl proteinases	Production of azelaic acid, lipases, and proteases, as well as the ability to evade host immune responses	Biofilm formation, Production of resistant pigments
Commonly used antifungals	Fluconazole, Echinocandins and Amphotericin B	Ketoconazole, Itraconazole, and Fluconazole	Fluconazole and Amphotericin B

## Data Availability

All data supporting the findings of this study are available within the paper.

## Abbreviations

PV: Pityriasis versicolor
AV: Acne vulgaris
PCR: Polymerase chain reaction
RFLP: Restriction Fragment Length Polymorphism
ITS: Internal Transcribed Spacer
MTCC: Microbial Type Culture Collection
mDA: Modified Dixon’s Agar
LPCB: Lactophenol cotton blue
DAPI: 4′,6-diamidino-2-phenylindole
IFI: Invasive Fungal Infections
AD: Atopic dermatitis
SD: Seborrheic dermatitis
PFGE: Pulsed-Field Gel Electrophoresis
RAPD: Random Amplified Polymorphic DNA analysis
AFLP: Amplified Fragment Length Polymorphism
DGGE: Denaturing Gradient Gel Electrophoresis
NGS: Next-generation sequencing
